# Determinants of Cervical Cancer Screening Uptake among Women in Ilorin, North Central Nigeria: A Community-Based Study

**DOI:** 10.1155/2016/6469240

**Published:** 2016-01-06

**Authors:** Ajibola Idowu, Samuel Anu Olowookere, Aderonke Tolulope Fagbemi, Olumuyiwa Ayotunde Ogunlaja

**Affiliations:** ^1^Department of Community Medicine, Faculty of Clinical Sciences, Bowen University Teaching Hospital, P.O. BOX 15, Ogbomoso 210251, Oyo State, Nigeria; ^2^Department of Community Health, Obafemi Awolowo University Teaching Hospital, Ile-Ife 220001, Osun State, Nigeria; ^3^Department of Community Health, Ondo State Specialist Hospital, PMB 603, Ondo State, Nigeria; ^4^Department of Obstetrics and Gynaecology, Bowen University Teaching Hospital, P.O. BOX 15, Ogbomoso 210251, Nigeria

## Abstract

*Introduction*. Cancer of the cervix is the leading cause of cancer deaths among women in developing countries. Screening is one of the most cost effective control strategies for the disease. This study assessed the determinants of cervical cancer screening uptake among Nigerian women.* Methodology*. This cross-sectional study was conducted using multistage sampling technique among 338 participants in Ilorin, North Central Nigeria. A pretested questionnaire was used for data collection and data analysis was done using SPSS version 21. Chi-square test was used for bivariate analysis while binary logistic regression was used for multivariate analysis. Statistical significance was set at *p* < 0.05.* Results*. Only 8.0% of the respondents had ever been screened for cancer of the cervix. The proportion of women who had ever been screened was significantly higher among those who demonstrated positive attitude to screening (81.5%, *p* = 0.001), respondents who were aware of the disease (100.0%, *p* = 0.001), and those who were aware of cervical cancer screening (88.9%, *p* = 0.001). Respondents who had negative attitude had 63% lesser odds of being screened compared to those who had positive attitudes towards screening (AOR; 0.37, 95% CI; 0.01–0.28).* Conclusion*. There is urgent need to improve the knowledge base and attitude of Nigerian women to enhance cervical cancer screening uptake among them.

## 1. Introduction

Cancer of the cervix is currently the commonest cancer and the leading cause of cancer deaths among women in developing countries [1]. It is also the second most common cancer among women worldwide [2]. In 2008 alone, not less than 530,000 new cases of the disease and 275,000 deaths were recorded globally. Surprisingly, 90% of these deaths were recorded in the developing countries. In the WHO African region, about 75,000 new cases were recorded for the same year [1]. In Nigeria, an estimated 10,000 new cases of cervical cancer and 8000 deaths due to the disease are recorded among women yearly [3]. Moreover, Nigeria has an estimated five-year prevalence of 21.6% for cervical cancer as published in GLOBOCAN fact sheets of 2012 [4]. In 2012, Durowade et al. reported 5% as the prevalence of cervical cancer among women in Ilorin [5]. However, Ijaiya et al., in 2004, showed that cancer of the cervix accounted for 63% of all gynaecological cancers seen in the Obstetrics and Gynaecology Department of the University of Ilorin Teaching Hospital, Ilorin, Nigeria [6].

Papanicolaou (Pap) smear cytology screening method to identify precancerous lesions has helped in achieving massive reduction in the burden of cancer of the cervix especially in the developed countries [7, 8]. Other less invasive techniques have been developed for rapid screening of cancer of the cervix. Such techniques include Visual Inspection with Acetic Acid (VIA) and Visual Inspection with Lugol's Iodine (VILI). Although these methods are faster and less cumbersome, they have been discovered to be less sensitive compared to cytologic examination through Pap smear. VIA in particular has been associated with high false positive results leading to immense psychological problems and wrong treatment of affected women [9].

Due to the increasing burden of cancers generally, the World Health Assembly (WHA), in 2005, adopted resolution 58.22 which urged member states to intensify action against cancer through creation of National Cancer Control Programmes [10]. In Nigeria, the National Cancer Control Programme was developed in 2008 with the view of reducing the morbidity and mortality associated with cancer and its socioeconomic impacts. Within the framework of the National Cancer Control Plan, the Federal Ministry of Health (FMOH) established a cervical cancer control plan. The plan adopted screening for early disease detection of cervical cancer and human papilloma virus (HPV) vaccination for primary prevention in girls of 9–15 years [11]. The level of implementation of this plan is still debatable in Nigeria.

Although screening is a known cost effective strategy used in reducing the burden of cervical cancer worldwide, its uptake particularly in developing countries is still abysmal [12, 13]. One of the barriers to access is that most cervical cancer screening services (provided by governmental and nongovernmental agencies) in Nigeria had been sporadic and poorly coordinated. Most services are urban-based; the rural and semiurban dwellers are often neglected. Another problem is low awareness of women about cancer of the cervix and cervical cancer screening. Such cases are seen at their advanced stages when physicians could do nothing to cure them.

In Ilorin, accessibility to cervical cancer screening services has been questionable. Available screening services are mostly found in government owned tertiary and secondary health facilities with assistants from few nongovernmental organizations. The cost of screening could be as high as five thousand naira (25 USD) in such facilities. In a country such as Nigeria, with a timid population of people living below the poverty line and with a healthcare system that is predominantly dependent on out-of-pocket expenditure, such cost of service could be prohibitive. However, cytological screening using Pap smear seems to be the preferred method of screening in these facilities.

In spite of efforts from governmental and nongovernmental organizations to improve access to cervical cancer screening services in Nigeria, uptake has been appalling. Several studies have documented factors associated with uptake of cervical screening tests worldwide. Such factors include age of the women, their marital status, parity, risk perception, financial constraint, and knowing someone who has cancer of the cervix [14, 15]. It is therefore vital to understand contextually how some of these factors influence uptake of cervical cancer screening exercise among Nigerian women. This study aimed at providing information that could be useful to policy makers in shaping cervical cancer screening programmes in Nigeria. It also aimed at bridging the practice gap for cervical screening among Nigerian women.

The study objectives were to assess women awareness level, their knowledge about cervical cancer and cervical cancer screening, and their attitudes to cervical cancer screening. It also sought to identify factors influencing uptake of screening programmes among women residing in Ilorin, Kwara State.

## 2. Materials and Methods

### 2.1. Study Site

The study was conducted in Ilorin West Local Government Area (LGA), Kwara state of Nigeria. This LGA is predominantly urban according to the 2006 National Population Result for Nigeria. The LGA is regarded today as the premier local council in the state not only because it has historical antecedent but because it hosts the headquarters of the emirate councils. The administrative headquarters of the LGA are at Oja Oba. According to the 2006 population figure, the LGA has about 364, 666 people, with women constituting 47.4% [16]. The inhabitants of the LGA have different religious affiliations such as Islam, Christianity, and Traditional religions. The predominant occupations of the inhabitants of the town include farming and trading but sizeable proportion of the people are civil servants.

### 2.2. Study Design

The study employed community-based, cross-sectional descriptive design.

### 2.3. Participants and Sampling

The sample size was calculated using the Leslie Kish formula for estimating single proportion [17]. Based on documentation of previous study conducted in Nigeria by Nwozor and Oragudosi [18], a proportion of 36% was used as the percentage of Nigeria women who were aware of cancer of the cervix. A precision of 5% was used and correction for nonresponse was made. Thus, a total of 338 women were selected using multistage sampling technique between May and June 2015. The first stage involved selection of an enumeration area from the eight enumeration areas in the LGA by balloting. In the second stage, two communities in the selected enumeration area were selected by balloting. All households in the chosen communities with eligible respondents were selected for the study. In houses where there are more than one household with eligible respondents, a household was randomly selected by balloting.

### 2.4. Inclusion Criteria

All adult women who were at least 21 years of age [19] and who gave their written consents were recruited to participate in the study.

### 2.5. Exclusion Criteria

Women who were too ill or not consenting to participate were exempted from the study.

### 2.6. Study Procedures

Data were obtained using semistructured interviewer guided questionnaire. The questionnaire was developed based on information obtained from previous studies on cervical cancer screening. Data were collected on sociodemographic characteristic of the respondents, their awareness on cancer of the cervix/screening, knowledge about the disease, their attitudes towards screening, and reasons for nonscreening. Respondents who had not been screened were asked to select one best reason (from a list of options) for their lack of screening. The questionnaire was translated to Yoruba language and back-translated to English language for Yoruba speaking respondents. The back translation was done to retain the original meaning of the questions asked. The questionnaire was pretested in another enumeration area different from the one selected for the main study. Ambiguous questions observed during pretesting were either rephrased or removed in line with the study objectives. Five research assistants were recruited and trained for the purpose of data collection.

### 2.7. Data Analysis

The data were field-edited daily and Statistical Package for Social Sciences (SPSS, version 21) was used for analysis. Initial analyses were done by generating frequency tables and graphs while further analyses were done to explore statistical association between variables. Appropriate bivariate analysis was carried out to assess statistical association depending on variable types and a stepwise logistic regression model was performed to identify factors that were significantly associated with uptake of cervical cancer screening. Independent variables in the model were selected based on whether they were significant at bivariate level and/or on whether they had been reported in literatures as significant predictors of uptake of cervical screening. Some of the independent variables used include age of the respondents, socioeconomic class, and marital status. The level of statistical significance was set at *p* value <0.05. Control for potential confounders such as age and socioeconomic class of respondents was done by placing our respondents in different categories. The adjusted odds ratio and 95% confidence interval were obtained to determine factors that were significantly associated with uptake of cervical cancer screening programmes among our respondents.

## 3. Key Variables and Measurements

### 3.1. Respondents' Awareness about Cancer of the Cervix and Cervical Cancer Screening

To assess this, respondents were asked if they had heard about cancer of the cervix and also about cervical cancer screening. The response to each of the questions was “yes” or “no.” Those whose responses were “yes” to either one or both questions were further asked about the sources of their information. Moreover, respondents were asked if they were aware of the benefits of cervical cancer screening. The response was also “yes” or “no.”

### 3.2. Respondents' Knowledge on Cancer of the Cervix

Three questions were asked on common symptoms of cancer of the cervix, four questions on common risk factors, and three questions on its prevention. The responses were scored and summed. Each was given a score of one for correct answers and zero for incorrect answers. The cut-offs were defined based on previous knowledge studies using mean scores. Respondents were rated over ten; respondents who scored between 0 and 4 points were categorized as having poor knowledge, those who scored 5–7 points were grouped as having fair knowledge, and those who scored 8–10 points were classified as having good knowledge of cervical cancer.

### 3.3. Respondents' Attitude towards Cervical Cancer Screening

Respondents were asked if they were willing to attend cervical cancer screening exercise. Those whose responses were “yes” were categorized as demonstrating positive attitude, while those who answered “no” were classified as showing negative attitude to screening.

### 3.4. Respondents' Social Class

Using Oyedeji's classification of social class [20], respondents' socioeconomic status was classified into three: low, middle, and high. This classification used a composite score of respondents' educational levels and occupational types of their spouses. Educational levels of respondents as well as occupational types of their spouses were scored. The score ranged from 1 to 5 for educational level. A score of 1 stood for respondents who could barely read or write or were illiterates, while a score of 5 was for those with university education or its equivalent. For spousal occupational types, the score also ranged from 1 to 5 with 1 standing for the unemployed, full-time housewives, and students and 5 standing for professionals such as doctors, lawyers, and engineers. Respondent's scores from each of the occupational and educational categories were added together and rated over 10. Those who scored less than 5 points were grouped into lower social class, those who scored from 5 to 7 points were grouped into middle social class, and those who scored between 8 and 10 points were grouped into high social class.

### 3.5. Ethical Consideration

Ethical approval for this study was sought from Bowen University Teaching Hospital's Research and Ethics Committee. Written informed consents were obtained from all respondents. Participation of women was also voluntary and their confidentiality was guaranteed by making the questionnaire anonymous: names of respondents were not requested in completing the questionnaire. Also data obtained were saved in a passworded computer. Eligible respondents who had not gone for screening as at the time of the survey or who were showing symptoms of cervical cancer were counseled and referred to University of Ilorin Teaching Hospital for immediate care.

## 4. Results

Out of the three hundred and sixty questionnaires distributed, 338 were returned satisfactorily completed, giving the response rate of 94%.  Table 1 revealed that the mean age of the women who participated in the study was 30 ± 8 years. More than three-quarters (88.8%) of them were in the 21–35-year age range. Two hundred and twenty seven (67.2%) of them were married; most (43.2% and 36.7%) were in the lower and middle socioeconomic classes, respectively. Majority (68.0%) of the respondents practiced Islam and were resident within Ilorin Township (79.0%).

**Figure 1:**
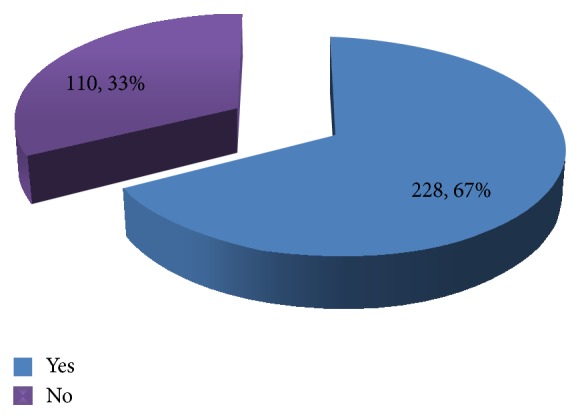
Awareness of respondents about cervical cancer.

**Figure 2:**
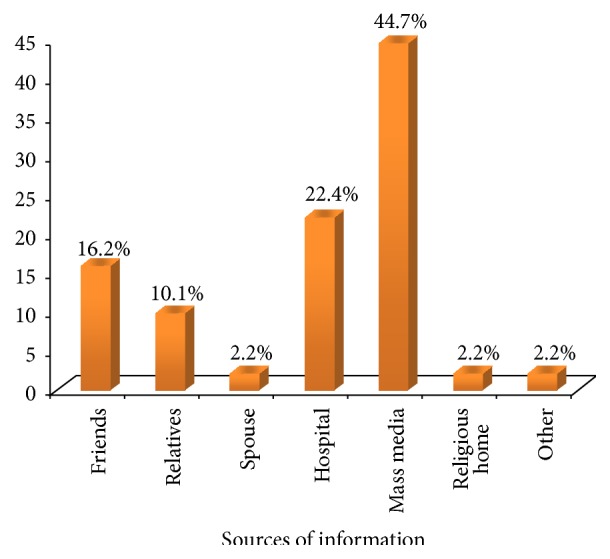
Respondents' sources of information about cervical cancer.

**Figure 3:**
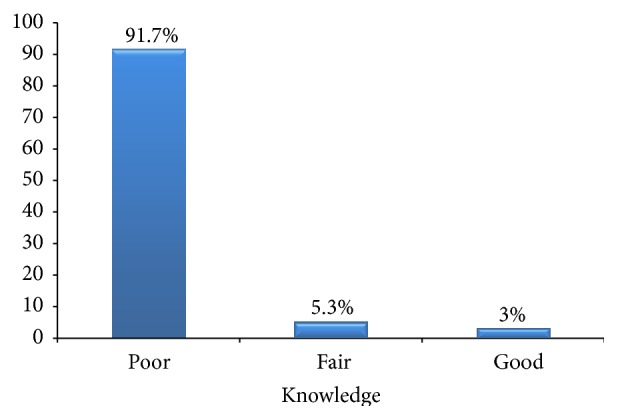
Knowledge of respondents on cancer of the cervix.

As shown in Figures 1, 2, and 3, most (67%) of the respondents had heard about cancer of the cervix. Mass media were the commonness sources of information, reported by 102 (44.7%) of the 228 of the women who were aware of the disease. Meanwhile, 92.0% of the respondents demonstrated poor knowledge on cancer of the cervix.

Most of the interviewees (67%) were aware of cervical cancer screening, also 66.9% of them were aware of the benefits of screening in cervical cancer disease control (Table 2). Moreover, majority (97.0%) of the respondents had positive attitude to cervical cancer screening. However, only 27 (8.0%) of the respondents had ever been screened for the disease. Eight (29.6%) of such women claimed that they had been screened twice, while 25.9% of them said that they had been screened on three occasions. Low risk perception regarding cancer of the cervix was the commonest reason for not participating in screening activities among respondents who had never been screened before; this was reported by 36.3% of such women (Table 2).

At the bivariate level (Table 3), the proportion (45.5%) of women who had ever been screened for cancer of the cervix was significantly higher among respondents who had positive attitude towards screening compared to those who had negative attitude (*p* = 0.001). The proportion (12.0%) was also significantly higher among those who were aware of cancer of the cervix (*p* = 0.001) as well as those who were aware of cervical cancer screening (11.2%, *p* = 0.001).

The results of multivariate analysis are presented in Table 4; only respondents' attitude towards screening for cancer of the cervix was found to be a significant predictor of screening uptake. Women who had negative attitude towards screening had 63% less odds of being screened compared to those who had positive attitude. This was found to be statistically significant (AOR; 0.37, 95% CI; 0.05–0.279).

## 5. Discussion

Almost three-quarters of our respondents were aware of cancer of the cervix. This did not however translate to good knowledge as 92% of the women demonstrated poor knowledge on the disease. Our finding compares with what had been reported in similar studies around the world. For instance, a study conducted among Gabonese women by Assoumou et al. revealed that 91.6% of the respondents had heard about cancer of the cervix [21]. Also, another study conducted among women in Bangladesh in 2014 by Ferdous et al. revealed that only 12% of the respondents had good knowledge of cervical cancer [22]. In Nigeria, however, some studies had reported slightly lower figures as the proportion of women who are aware of cervical cancer. For example, Wright et al. reported in 2014 that only 37.2% of the women interviewed had heard about cancer of the cervix [23]. The differences in figures could be due to differences in demographic characteristics of the participants in the two studies; while the current study was conducted among female participants only, the Lagos study included male participants. Male subjects are not likely to be as informative with regards to cervical cancer as women. A similar study conducted by Akinola et al. in Nigeria also revealed that only 47.1% of the women interviewed had heard about cervical cancer, while 39.5% of them knew something about Pap smear [24]. This study was hospital-based and may not be representative of the true awareness level of women in the community as opposed to the current study that was community-based.

The current study revealed that only 8.0% of the respondents had ever done Pap smear test before. Low risk perception was the main reason attributed to nonscreening of most of the respondents who had never been screened. This finding is in keeping with what literatures had reported in different parts of the world. For instance, a study by Singh et al. among women visiting tertiary care in Delhi in 2014 shows that only 7.3% of the women interviewed had ever done Pap smear test before [25]. Moreover, Shivanthan et al. reported that only 18.1% of the respondents in Sri Lanka had ever had a Pap smear test [26]. Similarly, Karadag et al. in a study among Turkish women reported that 73% of the respondents had never been screened for cancer of the cervix before [27]. In Nigeria, Wright et al. reported that only 5.1% of women in Lagos had ever undergone Pap smear testing [23]. However, Assoumou et al. reported that 65.1% of the women interviewed in Gabon had gone for Pap smear test before [21]. The reason for higher figure in the Gabonese study could have been due to a difference in the socioeconomic characteristics of the respondents; 63% of the respondents had university education and 51.6% were employed. In contrast, most of the respondents in the current study were in the lower socioeconomic class. This implies that most did not have university education and were not employed. Educated and employed people are expected to have better access to health information which could help them take appropriate health steps.

The observed low uptake of cervical cancer screening recorded in the current study could thus be attributable to such factors as low socioeconomic status of study participants. This is because educational and occupational status of people often determines their awareness level about a particular health condition and their financial capability to access healthcare services. The low uptake could also be due to poor availability of screening services within Ilorin West LGA and poor knowledge of people about the disease and its screening. About 20% of the women interviewed had never gone for screening because they did not know where to get the services. Moreover, cervical cancer screening services are mostly available in tertiary health institutions with catastrophic cost implications in most cases. In fact, not less than 13.5% of our respondents said that they could not access screening due to cost considerations. Also, a sizable proportion (36.3%) of them did not go for screening because of low risk perception. “Perceived risk” has been documented as a key determinant of health behavior of people [28]. In fact, McCaul et al. found a positive association between risk perception and uptake of screening for certain cancers [29]. Hence, low risk perception could have resulted in false assurances among our respondents culminating into low screening uptake among them. Husband refusal and fear of being tagged promiscuous were other reasons that could also explain the low screening uptake among our respondents. In countries with strong cultural values and family ties as Nigeria, husbands are the key decision takers in most homes. Thus, women are often careful of services requested from healthcare providers in order not to be tagged as women of low virtues by their spouses and their significant others. Moreover, almost 20.0% of the women who were interviewed could not go for screening for fear of positive result after screening. Vrinten et al. showed that thought of positive results after screening predicted low uptake of colorectal cancer screening among respondents in the United Kingdom [30]. This could have also been responsible for the low cervical cancer screening observed among our respondents

Meanwhile, the current study revealed that respondents with negative attitudes towards cervical cancer screening were the least likely to have been screened. However, other sociodemographic variables were not significantly associated with uptake of Pap smear screening test among our respondents as documented in various literatures.

## 6. Study Limitation

This study was conducted in urban communities; the result may not be generalizable to rural dwellers due to prevailing rural-urban disparity in the socioeconomic conditions of people in Nigeria. The fact that the study was community-based helped in increasing its external validity.

## 7. Conclusion

Most women in North Central Nigeria demonstrated poor knowledge about cervical cancer and low uptake of cervical cancer screening. Since early case detection through screening is the most cost effective activity for reducing the morbidity and mortality from cancer of the cervix, reproductive health experts and policy makers need to demonstrate more commitment in creating awareness about cervical cancer. They also need to make screening tests available at affordable costs through the establishment of more screening centers in the North Central geopolitical zone in particular and in Nigeria as a whole. The existing screening programmes which are majorly from nongovernmental organizations and largely in urban areas need to be decentralized and harmonized for greater efficiency. Also, there is need to integrate cervical cancer screening exercise into the mainstream healthcare services in the hospitals. Women who are at least 21 years of age particularly those with family history of cervical cancer must be encouraged to opt for cervical cancer screening at every available opportunity. Finally, there is need to increase the number of healthcare workers with requisite skills to conduct cervical cancer screening in Nigeria.

## Figures and Tables

**Table 1:** Respondents' sociodemographic characteristics.

Variables	Frequency (%)
Age groups	
21–35	300 (88.8)
36–55	35 (10.4)
≥56	3 (0.9)
Mean ± SD	**30 ± 8**
Never married	96 (28.4)
Married	227 (67.2)
Separated	5 (1.4)
Divorced	2 (0.6)
Widowed	8 (2.4)
Social status	
Low	146 (43.2)
Middle	124 (36.7)
High	68 (20.1)
Religion	
Islam	230 (68.0)
Christianity	106 (31.4)
Traditional	2 (0.6)
Place of residence	
Ilorin	267 (79.0)
Outside Ilorin	49 (14.5)
Outside Kwara State	22 (6.5)

**Table 2:** Respondents' awareness, attitude, and uptake of cervical cancer screening.

Variables	*N* = 338 *n* (%)
*Aware of screening*	
Yes	228 (67)
No	110 (33)
*Aware of benefits of screening*	
Yes	226 (66.9)
No	112 (33.1)
*Attitude to screening*	
Positive	327 (97)
Negative	11 (3)
*Screening uptake*	
Yes	27 (8.0)
No	311 (92.0)
*Number of times*	*n = 27*
Once	7 (25.9)
Twice	8 (29.6)
Three or more times	7 (25.9)
Cannot remember	5 (18.5)
*Reasons for not screening*	*n = 311*
Low risk perception	113 (36.3)
Husband did not agree	7 (2.3)
Fear of being tagged promiscuous	38 (12.2)
High cost of screening	43 (13.5)
Fear of been diagnosed of cancer	18 (5.8)
Did not know where to go for screening	62 (19.9)
Others	31 (10.0)

**Table 3:** Association between respondents' characteristics and uptake of cervical cancer screening.

Variables	Uptake of cervical cancer screening		Total	*χ* ^2^	*p*
	Yes *N* = 27 *n* (%)	No *N* = 311 *n* (%)	*N* = 338 *n* (%)		
Age groups					
21–35	25 (8.3)	275 (91.7)	300 (89.0)		
36–55	2 (5.7)	33 (94.3)	35 (10.0)		
≥56	0 (0.0)	3 (100.0)	3 (1.0)	0.354^*∗∗*^	0.838
Marital status					
Never married	9 (9.4)	87 (90.6)	96 (28.4)		
Married	18 (8.0)	209 (92.0)	227 (67.1)		
Separated	0 (0.0)	5 (100.0)	5 (1.4)		
Divorced	0 (0.0)	2 (100.0)	2 (0.5)		
Widowed	0 (0.0)	8 (100.0)	8 (2.2)	0.954^*∗∗*^	0.917
Social status					
Low	7 (5.7)	117 (94.3)	124 (37.0)		
Middle	17 (11.7)	129 (88.3)	146 (43.0)		
High	3 (4.4)	65 (95.6)	68 (20.0)	4.764^*∗∗*^	0.092
Religion					
Islam	16 (7.0)	214 (93.0)	230 (68.0)		
Christianity	11 (10.4)	95 (89.6)	106 (31.0)		
Traditional	0 (0.0)	2 (100.0)	2 (1.0)	1.525^*∗∗*^	0.466
Attitude					
Unfavourable	5 (45.5)	6 (54.5)	7 (2.0)		
Favourable	22 (6.7)	305 (93.3)	327 (98.0)	21.715	0.001^**∗**^
Awareness of cervical cancer					
Yes	27 (12.0)	201 (88)	228 (68.0)		
No	0 (0.0)	110 (100.0)	110 (32.0)	14.157	0.001^**∗**^
Awareness of screening					
Yes	24 (11.2)	190 (88.8)	214 (63.0)		
No	1 (1.4)	69 (98.6)	70 (21.0)		
Do not know	2 (3.7)	52 (96.3)	54 (16.0)	8.478^*∗∗*^	0.014^**∗**^
Knowledge on cancer of the cervix					
Good	27 (8.7)	283 (91.3)	310 (92.0)		
Fair	0 (0.0)	18 (100.0)	18 (5.0)		
Poor	0 (0.0)	10 (100.0)	10 (3.0)	2.650^*∗∗*^	0.266

^*∗*^Statistically significant at *p* < 0.05. ^*∗∗*^Likelihood Chi-square test used.

**Table 4:** Determinants of uptake of cervical cancer screening among the respondents.

Variable	AOR	*p* value	95% CI
Age			
21–35 (RC)	1		
36–55	0.772	0.816	0.088–6.810
≥56	0.682	0.693	0.102–4.552
Socioeconomic class			
Low (RC)	1		
Middle	0.817	0.787	0.189–3.534
High	0.309	0.075	0.085–1.126
Attitude to screening			
Positive (RC)	1		
Negative	0.37	0.001	0.005–0.279^*∗*^

^*∗*^Statistically significant at *p* < 0.05; RC: reference category; AOR: adjusted odds ratio; CI: confidence interval.
